# A prominent and conserved role for YY1 in Xist transcriptional activation

**DOI:** 10.1186/1756-8935-6-S1-P62

**Published:** 2013-03-18

**Authors:** Jean-Francois Ouimette, Mélanie Makhlouf, Andrew Oldfield, Pablo Navarro, Claire Rougeulle

**Affiliations:** 1 CNRS, UMR7216 Epigenetics and Cell Fate, F-75013 Paris, France; 2 Paris Diderot University, Sorbonne Paris Cité, F-75013 Paris, France; 3 MRC Centre for Regenerative Medicine, School of Biological Sciences, University of Edinburgh, 5 Little France Drive, Edinburgh EH164UU, Scotland, UK

## 

Dosage compensation for X-linked genes in female mammals relies on X-chromosome inactivation. This process involves monoallelic up-regulation of the non-coding *Xist* RNA which coats in *cis* the chromosome and triggers epigenetic reprogramming that will prevent chromosome-wide transcription. *Xist* transcription is controlled, directly and/or indirectly, by several ncRNA (*Tsix*, *Jpx*, *Ftx*) and factors (Sox2, Oct4, Nanog, Rex1, Rnf12). However, mechanisms leading to *Xist* monoallelic regulation remain poorly understood.

Using mouse ES and differentiating cells, we identified specific YY1 and CTCF interacting sites on the *Xist* locus of the inactive X-chromosome. We show that this monoallelic binding is controlled by DNA methylation. To gain further insights into YY1 functional role on the Xist locus, we conducted YY1 knockdown experiments. We show by RNA-FISH that depletion of YY1 in female somatic cells impairs the accumulation of *Xist* on the inactive X-chromosome. This is likely due to a transcriptional effect as we observe a drastic reduction of both spliced and unspliced *Xist* RNA levels. This hypothesis is further reinforced by the *in vitro* analysis *Xist* promoter activity, which displays strict dependency on the YY1 binding sites. Importantly, YY1 is also necessary for the upregulation of *Xist* that triggers X-chromosome inactivation. Taken together, these results suggest a strong requirement for YY1 in the upregulation and maintenance of *Xist* transcription. Importantly, we demonstrate that the function of YY1 in the control of *Xist* expression is conserved in humans and predicted in other mammalian species.

These results highlight the importance of YY1 both in the monoallelic upregulation of *Xist* at the exit of pluripotency and in the maintenance of its expression in somatic cells. Taken together with previous studies, we propose that through its dual action on *Tsix* and *Xist*, YY1 acts as a bimodal transcriptional regulator of X-inactivation.

